# Severe COVID-19 Pneumonia in an Unvaccinated Female Treated With Remdesivir

**DOI:** 10.7759/cureus.30286

**Published:** 2022-10-14

**Authors:** Kaylee Ortega, Andrew George, Charlotte R DeGeorge, Thor S Stead, Rohan Mangal, Jesse DeLosSantos, Latha Ganti

**Affiliations:** 1 Department of Biology, Trinity Preparatory School, Winter Park, USA; 2 Department of Pathology and Laboratory Medicine, Division of Biology and Medicine, Brown University, Providence, USA; 3 Department of Medicine, The Warren Alpert Medical School of Brown University, Providence, USA; 4 Department of Medicine, University of Miami Miller School of Medicine, Miami, USA; 5 Department of Emergency Medicine, Lakeland Regional Health Medical Center, Lakeland, USA; 6 Department of Emergency Medicine, HCA Florida Ocala Hospital, Ocala, USA; 7 Department of Emergency Medicine, Envision Physician Services, Nashville, USA; 8 Department of Emergency Medicine, University of Central Florida College of Medicine, Orlando, USA

**Keywords:** pneumonia, coronavirus disease 2019, ground glass opacity, obesity, covid-19 and obesity, remdesivir, peripheral ground glass opacities, covid-19 vaccine hesitancy, covid-19

## Abstract

Though recent developments in the management of the coronavirus disease 2019 (COVID-19) pandemic have resulted in significant progress, its continued persistence demands continued consideration both of larger scale public health factors as well as individual patient management. We present a case that provides a broad perspective across several issues within both categories, of a morbidly obese 34-year-old unvaccinated female presenting with respiratory distress secondary to COVID-19 pneumonia, managed through remdesivir therapy. Though this case presents an example of successful management, it nonetheless emphasizes the demand for a renewed focus on vaccine hesitancy and obesity as public health issues, particularly within the context of the pandemic.

## Introduction

Caused by severe acute respiratory syndrome coronavirus 2 (SARS-CoV-2), the highly contagious infectious disease coronavirus disease 2019 (COVID-19) has resulted in more than 6 million deaths worldwide as of March 2022. As the most consequential global health crisis since the influenza pandemic of 1918, COVID-19 was designated as a global pandemic by the World Health Organization (WHO) on March 11, 2020. The past 2.5 years have resulted in the development of novel therapeutics and vaccine development at unprecedented speed. The most important measure to prevent and decrease the transmission of SARS-CoV-2 is shown to be through vaccination [1]. To date, four vaccines have been authorized for emergency use or are approved by the FDA. In randomized placebo-controlled phase III trials, the BNT162b2 (BioNTech/Pfizer), mRNA-1273 (Moderna), and Ad26.COV2.S (Johnson & Johnson/Janssen) vaccines showed 95%, 94%, and 67% efficacy against symptomatic disease due to SARS-CoV-2 [2].

Advanced age and comorbidities such as obesity, chronic lung disease, and cardiovascular disease are risk factors for the development of severe COVID-19 and its associated complications. A common complication of severe COVID-19 illness is progressive or sudden clinical deterioration leading to acute respiratory failure and acute respiratory distress syndrome (ARDS). Currently, therapeutic options for COVID-19 include antiviral drugs, anti-SARS-CoV-2 monoclonal antibodies, anti-inflammatory drugs, and immunomodulatory agents available under FDA-issued Emergency Use Authorization (EUA). Remdesivir (a broad-spectrum antiviral agent) was approved by the FDA for clinical use to treat hospitalized patients with COVID-19 and was recently shown to produce an 87% lower risk of hospitalization or death than placebo when at-risk nonhospitalized patients with COVID-19 were treated with a three-day course of remdesivir in a randomized double-blind placebo-controlled trial [3].

Although advances in treatment and vaccine development have improved, lagging vaccination rates have contributed to the lack of transition from pandemic to endemic. As cases continue to persist, we must globally utilize effective vaccines, anti-viral medications, and diagnostic tests, and develop vaccines with protection against a broader range of variants. To mitigate continued severe COVID-19 cases with associated complications, vaccination must remain a continued public health effort. We present a case of a morbidly obese 34-year-old unvaccinated female presenting with respiratory distress secondary to COVID-19 pneumonia, managed through remdesivir therapy.

## Case presentation

A 34-year-old morbidly obese female presented to the ED by ambulance with a chief complaint of shortness of breath, with a recent history of gastrointestinal symptoms including diarrhea over the previous five days. She had previously visited the ED during the course of these symptoms suspecting possible COVID-19 but had tested negative at the time. She denied a history of alcoholism, recreational drug use, or asthma. She was unvaccinated against COVID-19 and stated she had no intention of ever receiving it.

Prehospital vital signs taken by emergency medical services (EMS) were significant for a low pulse oximetry oxygen saturation of 90% on room air. Four milligrams of ondansetron and albuterol nebulizer treatment were administered in the field by EMS.

Upon presentation, the patient was febrile with a temperature of 100.3ºF. Additional vitals at this time include a blood pressure of 139/78 mmHg (mean arterial pressure: 98 mmHg), pulse rate of 105, and respiratory rate of 24 breaths per minute. A focused physical examination confirmed a patent airway and moderate respiratory distress with accompanying stridor on auscultation. Physical examination did not otherwise reveal additional signs or symptoms, and the patient remained fully alert and oriented. She was given 125 mg methylprednisolone intravenously and started on bilevel positive airway pressure with a positive end-expiratory pressure of 10 cmH20 and pressure support of 5. She received acetaminophen for her fever and a sepsis protocol was initiated.

**Figure 1:**
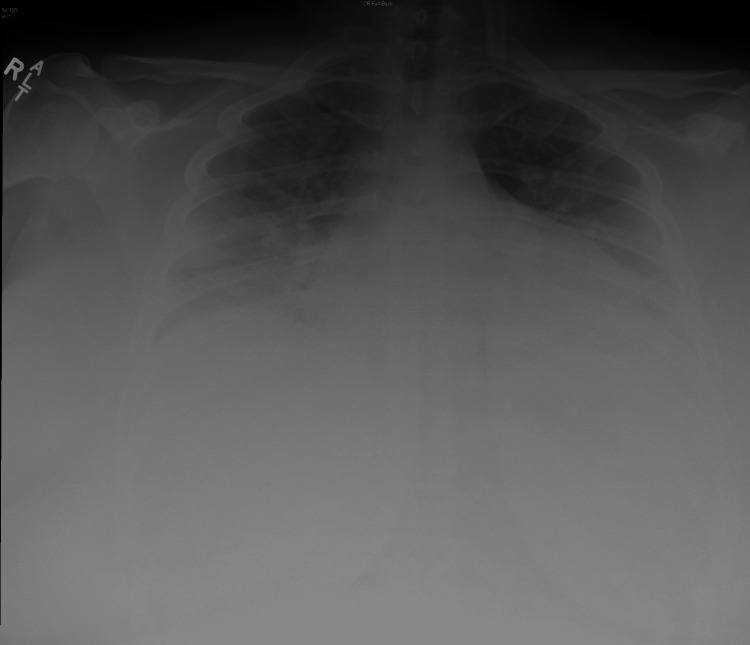
Chest radiograph on the day of ER visit demonstrating bilateral infiltrates and pneumonia

**Figure 2:**
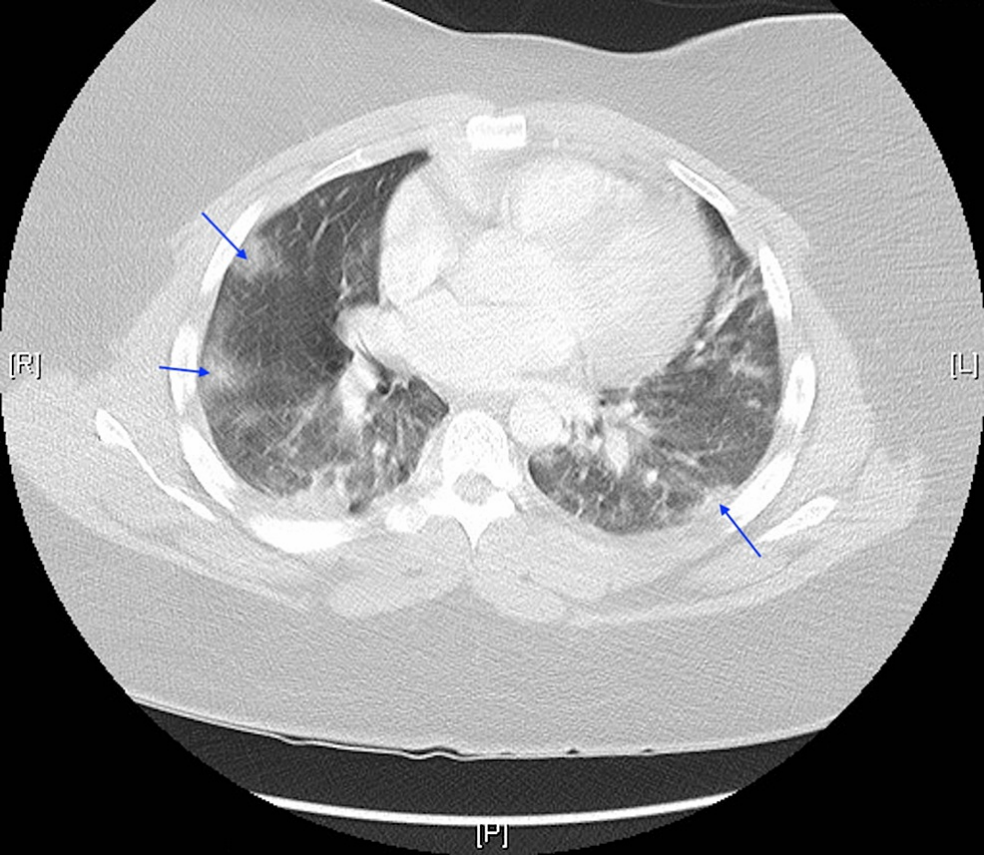
Chest CT demonstrating the classic ground glass opacities seen with COVID-19 infection

D-dimer elevated at 527 ng/mL FEU. The patient tested positive for COVID-19 through nucleic acid amplification and was diagnosed with acute respiratory distress secondary to COVID-19 pneumonia. The radiological examination included a chest radiograph that demonstrated bilateral infiltrates and pneumonia (Figure 1) and a chest CT scan that demonstrated the classic ground glass opacities seen with COVID-19 infection (Figure 2).

**Figure 3:**
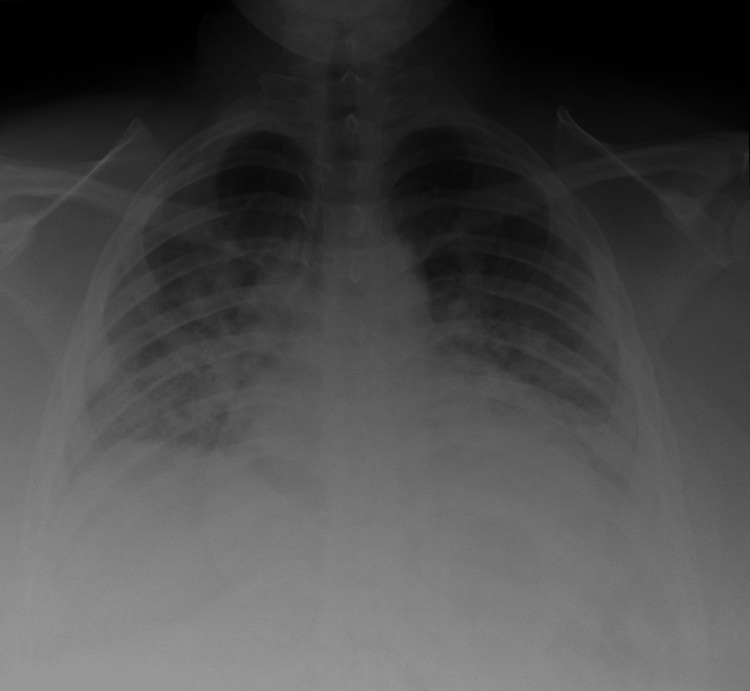
Chest radiograph two days later demonstrating marked improvement

The patient’s acute respiratory distress was stabilized for transfer to the main hospital, where she was started on remdesivir therapy per protocol. She improved steadily over the next two days. A repeat chest X-ray was performed, and a notable improvement could be seen (Figure 3).

After completing the remdesivir therapy, the patient was discharged home.

This case occurred during the Delta variant. The CDC vaccination recommendation at that time was two doses of vaccine and one booster for high-risk individuals. Vaccines were widely available during this time, so much so that out-of-state residents were welcomed to get vaccines as the supply was so available. This case occurred in Florida where there were (and still are) many unvaccinated individuals. At the time of the case, those getting very sick from COVID-19 were all mostly unvaccinated. Patients were asked about their vaccine reservations and time was spent by the involved clinicians dispelling vaccine myths. The only barrier to vaccines in this and other similar cases at the time was the misinformed disbelief of vaccine safety and efficacy.

## Discussion

Though the current state of the COVID-19 pandemic remains far superior compared with the pre-vaccine era, emerging research on acute complications, chronic long-term adverse events, and “brain fog” associated with contracting the disease reveals the need for continued disease vigilance [4,5]. In this regard, vaccine hesitancy remains a particularly concerning public health threat.

Indeed, vaccine hesitancy is not a particularly novel concept, but rather one that has become ubiquitous in the realms of public health, epidemiology, and infectious disease management summarizing various positions on vaccine administration ranging from directly opposed to cautiously in acquiescence. Its societal spread, especially in recent years, has brought about a drop in vaccination rates leading to a resurgence of vaccine-preventable disease (VPD) over the past half-decade to a degree previously unseen [6].

In the context of the COVID-19 pandemic, vaccine hesitancy has unsurprisingly manifested in a significant population of unvaccinated individuals. Despite all available vaccines in the US having achieved demonstrable and remarkable benchmarks in both individual and societal risk reduction, with a CDC-led report published earlier this year finding 14- and 53-fold higher risk of COVID-19 infection and death, respectively, in unvaccinated versus fully vaccinated persons, prevalent beliefs ranging from the skepticism of the pandemic severity (perhaps driven by case examples of relatively low-virulence or low acute severity COVID-19 infection) to mistrust of the vaccine itself persist [7-9]. Unfortunately, however, this persistence has resulted in avoidable disease spread among unvaccinated communities and families [10].

Interestingly, vaccine hesitancy presents even among healthcare workers despite their unique roles through the past years in managing the pandemic [11]. Vaccine hesitancy in such a population is uniquely alarming, given the implicit trust held by such individuals. Especially in the context of witnessed confirmation bias among vaccine-hesitant individuals, even a few examples of healthcare workers refusing or mistrusting existing vaccines may have a significant impact on those uncertain of their stance. Moreover, such examples provide continued validation for those campaigning against COVID-19 vaccination. Thankfully, an American Medical Association survey of practicing physicians shows that more than 96% have been fully vaccinated for COVID-19, with no significant difference in vaccination rates across regions [12].

As shown through the case we report, such vaccine hesitancy can result in emergent symptoms. Though management of patient condition was ultimately successful in this case, a more directed effort to address the problem of vaccine hesitancy both overall and within the context of the COVID-19 pandemic is needed.

The patient at hand additionally presented with a second risk factor in her morbid obesity. Patients suffering from obesity are more likely to suffer from respiratory infections due to mechanical and inflammatory issues [13]. Compared to historical non-COVID-19 controls, there are greater rates of obesity among COVID-19 patients [14]. Furthermore, in a recent study conducted in New York with a sample size of 5700 patients, there was a higher prevalence of obesity compared to diabetes in patients with COVID-19 [15]. Such findings are corroborated across studies originating from different countries, with 48% and 21-46% of COVID-19 patients being overweight or obese, respectively [16]. These facts indicate the status obesity holds as a risk factor for COVID-19.

The use of remdesivir therapy was indicated in this case and played a role in the successful management of the patient. Remdesivir is a nucleoside analog that became the first approved treatment for severe COVID-19 cases due to its broad anti-viral properties [17]. This treatment looked promising for severe COVID-19 cases when preliminary data showed that this medication fought SARS-CoV in a human cell line that was sensitive to the virus [18]. Remdesivir has now been shown to have a positive impact on cases involving ebolavirus, Middle East respiratory syndrome coronavirus (MERS-CoV), SARS-CoV, and SARS-CoV-2 [17]. A recent study found that remdesivir used on hospitalized COVID-19 patients lead to a recovery time of about 10 days compared to 15 days with patients in the placebo group [19]. The effectiveness of remdesivir therapy held true for our high-risk patient as well, and such therapies should continue to be considered in the management of severe COVID-19.

## Conclusions

In summary, we present a case that highlights several important aspects of the modern management of the COVID-19 pandemic and acute COVID-19 pneumonia and secondary respiratory distress. Firstly, continued overall progress against the disease demands a renewed emphasis on vaccine hesitancy and vaccine-hesitant individuals, with a growing need for further research into the motivations behind such viewpoints and holistic solutions. Secondly, obesity as a risk factor for COVID-19 presents another public health avenue into pandemic management. Finally, the use of remdesivir presents a promising emerging therapeutic approach for the management of COVID-19. Continued awareness of current topics in COVID-19 management remains critical, particularly as pandemic restrictions continue to loosen or expire.
